# Supplementary material for: Drug repurposing for aging research using model organisms
Source: Aging Cell. 2017 Jun 16;16(5):1006–15. doi: 10.1111/acel.12626 (PMC5595691; doi:10.1111/acel.12626)
Supplement: Supplementary file 7 — Data S1 Zip‐Archive of all report cards. [file ACEL-16-1006-s007.zip › RC_X8Z.pdf]

X8Z

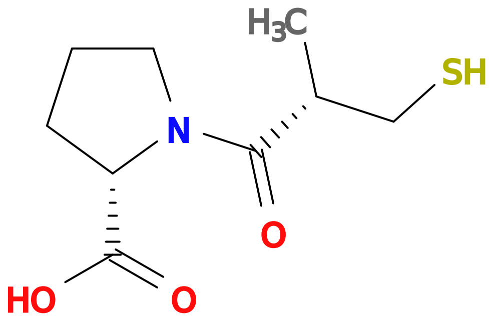

Database identifiers

ChEMBLCompound	CHEMBL1560
DrugBank	DB01197
CHEBI	3380
eMolecules	489049

Ranking

	Rank	Score
Drosophila	281/697	0.595
C. elegans	220/591	0.235

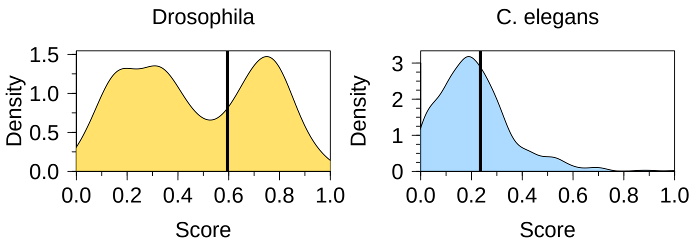

	Ageing implication	Domain conservation	Binding site conservation	Binding affinity	Bioavailability	Lipinski	Promiscuity	Purchasability	Drug approval	**Total**
Drosophila	0.476	0.982	1.0	0.94	(0.9)	0.0	-0.0	0.1	0.1	0.595
C. elegans	0.476	0.746	0.518	0.94	0.208	0.0	-0.0	0.1	0.1	0.235

Names

- **captopril**
- Capoten
- CP
- D-2-methyl-3-mercaptopropanoyl-L-proline
- D-3-mercapto-2-methylpropanoyl-L-proline
- Lopril
- Tensobon

Roles

EC 3.4.15.1 (peptidyl-dipeptidase A) inhibitor, antihypertensive agent, Angiotensin-converting enzyme inhibitor

Status

Approved drug (according to ChEMBL)	Yes
Administration Route	Oral
Number of Rule of 5 violations	0
Binding affinity to original target in log units (RF-Score prediction)	7.76
Burns *C. elegans* bioavailability prediction	-6.42

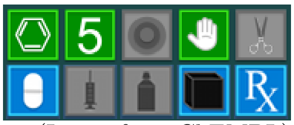

(Image from ChEMBL)

Medical Information

Indicatation: For the treatment of essential or renovascular hypertension (usually administered with other drugs, particularly thiazide diuretics). May be used to treat congestive heart failure in combination with other drugs (e.g. cardiac glycosides, diuretics, amp;beta;-adrenergic blockers). May improve survival in patients with left ventricular dysfunction following myocardial infarction. May be used to treat nephropathy, including diabetic nephropathy.
Mechanism of action: There are two isoforms of ACE: the somatic isoform, which exists as a glycoprotein comprised of a single polypeptide chain of 1277; and the testicular isoform, which has a lower molecular mass and is thought to play a role in sperm maturation and binding of sperm to the oviduct epithelium. Somatic ACE has two functionally active domains, N and C, which arise from tandem gene duplication. Although the two domains have high sequence similarity, they play distinct physiological roles. The C-domain is predominantly involved in blood pressure regulation while the N-domain plays a role in hematopoietic stem cell differentiation and proliferation. ACE inhibitors bind to and inhibit the activity of both domains, but have much greater affinity for and inhibitory activity against the C-domain. Captopril, one of the few ACE inhibitors that is not a prodrug, competes with ATI for binding to ACE and inhibits and enzymatic proteolysis of ATI to ATII. Decreasing ATII levels in the body decreases blood pressure by inhibiting the pressor effects of ATII as described in the Pharmacology section above. Captopril also causes an increase in plasma renin activity likely due to a loss of feedback inhibition mediated by ATII on the release of renin and/or stimulation of reflex mechanisms via baroreceptors. Captoprils affinity for ACE is approximately 30,000 times greater than that of ATI.
Toxicity: Symptoms of overdose include emesis and decreased blood pressure. Side effects include dose-dependent rash (usually maculopapular), taste alterations, hypotension, gastric irritation, cough, and angioedema.
Metabolism: Hepatic. Major metabolites are captopril-cysteine disulfide and the disulfide dimer of captopril. Metabolites may undergo reversible interconversion.
Retail: Advanced Pharmaceutical Services Inc., Amerisource Health Services Corp., Apotex Inc., Apotheca Inc., Apothecon, AQ Pharmaceuticals Inc. and 52 more suppliers
Apo-Capto 100 mg Tablet - 1.09 USD / tablet
Mylan-Captopril 100 mg Tablet - 1.09 USD / tablet
Novo-Captoril 100 mg Tablet - 1.09 USD / tablet
Nu-Capto 100 mg Tablet - 1.09 USD / tablet
Capoten 25 mg tablet - 1.13 USD / tablet
Capoten 50 mg tablet - 1.13 USD / tablet
and 20 more options

(Information from DrugBank)

Compound Target Characteristics

Angiotensin-converting enzyme

Best gene implication in ageing for this target family came from gene M0RB66 via mapping the annotation from Ensembl ENSRNOG00000007467 via mapping the annotation from RGD 2493 an-

notated in RGD 2014-03-11. Annotation GO 7568 (aging) was Inferred from Expression Pattern

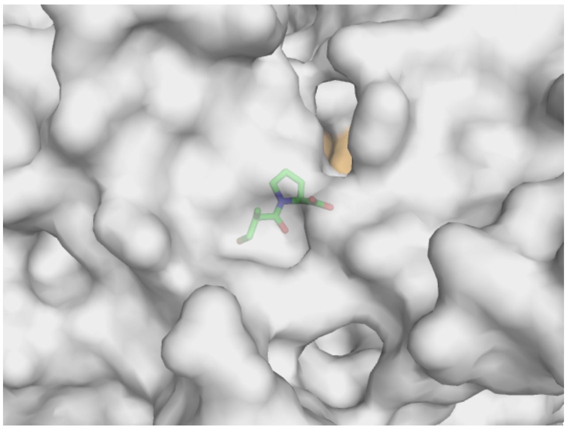

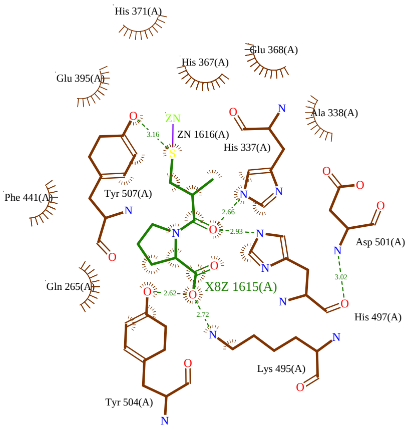

protein	amino acids contacts (binding site)
PDB:1j37:chainA:Q10714	Q H A H E H E F K H Y Y
PDB:2x8z:chainA:Q10714	Q H A H E H E F K H Y Y
tr:D3DU13:D3DU13_HUMAN	Q H A H E H E F K H Y Y
tr:L7MUH0:L7MUH0_HUMAN	Q H A H E H E F K H Y Y
sp:P12821:ACE_HUMAN	Q H A H E H E F K H Y Y
tr:M0RB66:M0RB66_RAT	Q H T H Q H E F K H Y Y
tr:Q8K233:Q8K233_MOUSE	Q H P H E H E F K H Y Y
sp:P09470:ACE_MOUSE	Q H P H E H E F K H Y Y
tr:Q3TU20:Q3TU20_MOUSE	Q H P H E H E F K H Y Y
sp:Q10714:ACE_DROME	Q H A H E H E F K H Y Y
sp:Q9VLJ6:ACER_DROME	Q H A H E H E F K H Y Y
sp:Q18581:ACN1_CAEEL	G H A S L Q D K H S A T

	whole protein		domain-based		contact-based	
protein	ident	simil	ident	simil	ident	simil
PDB:1j37:chainA:Q10714	0.98	0.99	0.99	1.0	1.0	1.0
PDB:2x8z:chainA:Q10714	1.0	1.0	1.0	1.0	1.0	1.0
tr:D3DU13:D3DU13_HUMAN	0.36	0.63	0.44	0.78	1.0	1.0
tr:L7MUH0:L7MUH0_HUMAN	0.35	0.65	0.45	0.8	1.0	1.0
sp:P12821:ACE_HUMAN	0.2	0.37	0.45	0.8	1.0	1.0
tr:M0RB66:M0RB66_RAT	0.31	0.64	0.4	0.79	0.83	0.93
tr:Q8K233:Q8K233_MOUSE	0.25	0.47	0.44	0.8	0.92	0.98
sp:P09470:ACE_MOUSE	0.19	0.36	0.44	0.8	0.92	0.98
tr:Q3TU20:Q3TU20_MOUSE	0.19	0.36	0.44	0.8	0.92	0.98
sp:Q10714:ACE_DROME	1.0	1.0	1.0	1.0	1.0	1.0
sp:Q9VLJ6:ACER_DROME	0.48	0.82	0.53	0.85	1.0	1.0
sp:Q18581:ACN1_CAEEL	0.16	0.49	0.23	0.71	0.17	0.52

Ance (FBgn0012037) associated phenotypes

partially lethal - majority live

(Information from FlyBase)

Ance (UniProt:Q10714) annotation

Function: May be involved in the specific maturation or degradation of a number of bioactive peptides. May play a role in the contractions of the heart, gut and testes, and in spermatid differentiation. (PubMed:12591244).

Cofactor: Zn(2+)Note=Binds 1 zinc ion per subunit.;

Enzyme regulation: Inhibited by captopril and, to a lesser extent, by lisinopril, trandolaprilat, fosinoprilat and enalaprilat. (PubMed:7775412, PubMed:8761461).

Biophysicochemical properties: Kinetic parameters: KM=33.5 uM for angiotensin I (PubMed:7775412, PubMed:8761461, PubMed:9839949); KM=53.4 uM for N-acetyl-Ser-Asp-Lys-Pro (PubMed:7775412,

PubMed:8761461, PubMed:9839949); KM=2.59 mM for Hip-His-Leu (PubMed:7775412, PubMed:8761461, PubMed:9839949); KM=10.26 mM for Hip-His-Leu-NH(2) (PubMed:7775412, PubMed:8761461, PubMed:9839949); KM=372 uM for (Leu5)enkephalin (PubMed:7775412, PubMed:8761461, PubMed:9839949); KM=1.88 mM for (Leu5)enkephalinamide (PubMed:7775412, PubMed:8761461, PubMed:9839949);
Subcellular location: Secreted, extracellular space.
Tissue specificity: Expressed in vesicular structures in spermatocytes and early spermatids (at protein level). (PubMed:12591244).
Developmental stage: Expressed in the amnioserosa during germ band elongation, shortening and heart morphogenesis. Expressed in midgut throughout embryogenesis.
Ptm: Glycosylated. (PubMed:8761461).
Disruption phenotype: Male flies are sterile. (PubMed:12591244).

(Information from UniProt)

Acer (FBgn0016122) associated phenotypes
circadian behavior defective, short lived, sleep defective

(Information from FlyBase)

Acer (UniProt:Q9VLJ6) annotation
Function: May be involved in the specific maturation or degradation of a number of bioactive peptides. May have a role in the specification of heart progenitors. (PubMed:12075344).
Cofactor: Zn(2+)Note=Binds 1 zinc ion per subunit. ;
Enzyme regulation: Inhibited by captopril, lisinopril, trandolaprilat, fosinoprilat and enalaprilat. (PubMed:9839949).
Biophysicochemical properties: Kinetic parameters: KM=1.2 mM for Hip-His-Leu (PubMed:9839949); KM=4.35 mM for Hip-His-Leu-NH(2) (PubMed:9839949); KM=40.6 uM for (Leu5)enkephalin (PubMed:9839949); KM=949 uM for (Leu5)enkephalinamide (PubMed:9839949); pH dependence: Optimum pH is 8.6. (PubMed:9839949);
Subcellular location: Secreted, extracellular space (PubMed:9839949).
Developmental stage: Expressed in presumptive heart cells during dorsal closure. (PubMed:8973330).
Ptm: Glycosylated. (PubMed:9839949).
Disruption phenotype: Defective heart morphogenesis leading to lethality. (PubMed:12075344).
Miscellaneous: In contrast to ance, does not hydrolyze angiotensin I.

(Information from UniProt)

acn-1 (WBGene00000039) associated phenotypes
embryonic lethal, lethal, sterile

(Information from WormBase)

acn-1 (UniProt:Q18581) annotation
Function: Required for larval molting, male tail development, and formation of adult alae. Inactive as a metallopeptidase, due to a lack of active site residues. (PubMed:14559923).
Tissue specificity: Expressed in embryonic and larval hypodermis, in the vulva during organogenesis, and in the ray papillae of the male tail. (PubMed:14559923).

(Information from UniProt)
